# *Trans*-Splicing Improvement by the Combined Application of Antisense Strategies

**DOI:** 10.3390/ijms16011179

**Published:** 2015-01-06

**Authors:** Ulrich Koller, Stefan Hainzl, Thomas Kocher, Clemens Hüttner, Alfred Klausegger, Christina Gruber, Elisabeth Mayr, Verena Wally, Johann W. Bauer, Eva M. Murauer

**Affiliations:** Department of Dermatology and EB House Austria, Paracelsus Medical University, Salzburg 5020, Austria; E-Mails: t.kocher@salk.at (T.K.); c.huettner@salk.at (C.H.); a.klausegger@salk.at (A.K.); c.gruber@salk.at (C.G.); el.mayr@salk.at (E.M.); v.wally@salk.at (V.W.); jo.bauer@salk.at (J.W.B.); e.murauer@salk.at (E.M.M.)

**Keywords:** RNA *trans*-splicing, antisense oligonucleotides, RNA repair, genetic diseases

## Abstract

Spliceosome-mediated RNA *trans*-splicing has become an emergent tool for the repair of mutated pre-mRNAs in the treatment of genetic diseases. RNA *trans*-splicing molecules (RTMs) are designed to induce a specific *trans*-splicing reaction via a binding domain for a respective target pre-mRNA region. A previously established reporter-based screening system allows us to analyze the impact of various factors on the RTM *trans*-splicing efficiency* in vitro*. Using this system, we are further able to investigate the potential of antisense RNAs (AS RNAs), presuming to improve the *trans*-splicing efficiency of a selected RTM, specific for intron 102 of *COL7A1*. Mutations in the *COL7A1* gene underlie the dystrophic subtype of the skin blistering disease epidermolysis bullosa (DEB). We have shown that co-transfections of the RTM and a selected AS RNA, interfering with competitive splicing elements on a *COL7A1*-minigene (*COL7A1*-MG), lead to a significant increase of the RNA *trans*-splicing efficiency. Thereby, accurate *trans*-splicing between the RTM and the *COL7A1*-MG is represented by the restoration of full-length green fluorescent protein GFP on mRNA and protein level. This mechanism can be crucial for the improvement of an RTM-mediated correction, especially in cases where a high *trans*-splicing efficiency is required.

## 1. Introduction

The RNA-based technology spliceosome-mediated RNA *trans*-splicing (SMaRT) has evolved into a potential tool to repair mutations at the mRNA level and has been successfully applied to various *in vitro* disease models including epidermolysis bullosa (EB), cystic fibrosis and hemophilia [1,2,3,4,5]. One of the most obvious advantages of SMaRT over gene replacement strategies is the size reduction of the transgene to deliver [6]. Therefore, the *COL7A1* gene is a condign target for SMaRT due to its huge size of more than 9 kb when transcribed into mRNA. The risk of genetic rearrangements caused by the abundant repetitive sequences of *COL7A1* [7] is significantly reduced by the insertion of size reduced fragments into the host genome. In principle, the *trans*-splicing reaction is facilitated by a constructed RNA *trans*-splicing molecule (RTM), which primarily base-pairs with a defined sequence on the target pre-mRNA of interest, thereby inducing the recombination of the two pre-mRNA molecules to a new chimeric mRNA. RNA *trans*-splicing can be used to reprogram 5', 3' or internal mRNA portions [6,8]. Its efficiency is mainly influenced by the splicing and binding characteristics of a designed RTM. The specificity of RNA *trans*-splicing is defined by the binding domain (BD), that base-pairs with the targetregion of choice. In recent studies we focused on the importance of highly efficient RTM BDs and we found that the length, binding localization and composition of the BD are the key factors for efficient *trans*-splicing [8,9,10,11]. We utilized a GFP-based cassette system to monitor the *trans*-splicing efficiency of randomly-designed RTMs on the mRNA and protein level [12].

In the present study we focus on further *trans*-acting factors apart from the BD, which improve the *trans*-splicing efficiencies of designed RTMs. In this context, we referred to the study of Coady and colleagues, in which they have demonstrated a way to increase the *trans*-splicing efficiency by reducing the competition of endogenous splice sites. Using a *cis-*splice site blocking antisense RNA molecules (AS RNAs), it was possible to enhance the *trans*-splicing efficiency in a spinal muscular atrophy (SMA) model system [13,14]. Currently there are no consensus rules for AS RNA design. In present studies [13,15], a series of overlapping AS RNAs of defined length (15–22 nts) were rationally synthesized for a defined target area. This is feasible for targeting a splice site within an intron-exon junction or a previously-identified splicing regulatory element (SRE).

However, the majority of SREs that regulate the splicing process are not known. For these reasons, we chose to exploit our established fluorescence-based RTM screening system in order to identify functional AS RNAs from a library of randomly generated AS RNAs that could increase the *trans*-splicing efficiency of a given RTM specific for the *COL7A1* gene, which is associated with the devastating skin disease dystrophic epidermolysis bullosa (DEB) [16,17]. We assume that the co-administration of a highly functional RTM together with an AS RNA, which interferes with competitive *cis*-splicing elements within the endogenous target pre-mRNA, further increases the repair efficiency to a sufficient level to overcome the effects of dominant mutations present in EB and other inherited diseases.

## 2. Results

### 2.1. Selection of an Efficient Binding Domain Specific for Intron 102 of COL7A1

**Figure 1:**
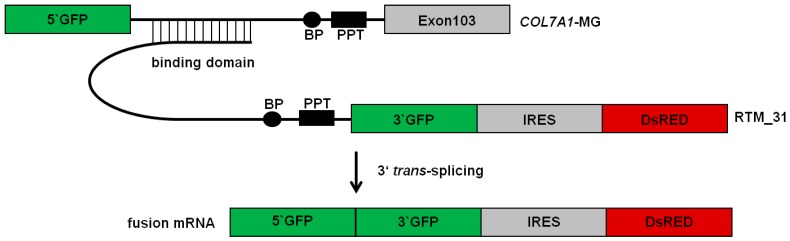
RNA *trans*-splicing molecules (RTMs) screening system and binding position of RTM_31 within *COL7A1* intron 102/exon 103. The fluorescence-based model system consists of a *COL7A1*-minigene (*COL7A1*-MG), carrying the 5' portion of GFP upstream of the RTM binding region intron 102/exon 103 of *COL7A1* and RNA *trans*-splicing molecules carrying a BD (binding domain) specific for intron 102 of *COL7A1*, a splicing domain (including branch point, BP; polypyrimidine tract, PPT; 3' acceptor splice site, ss), the missing portion of 3' GFP, an internal ribosomal entry site (IRES) and the reporter molecule, DsRED. Accurate *trans*-splicing between the *COL7A1*-MG and a functional RTM leads to fusion of both GFP parts provided by both interacting molecules, thereby restoring the function of the protein.

For the selection of a highly potent binding domain (BD) specific for the *COL7A1* target region, intron 102/exon 103 we used our recently published fluorescence-based screening system [10,11,12]. Using this method, a variety of BDs, specific for the *COL7A1* target region intron 102/exon 103, were cloned into the GFP-RTM, containing the 3' half of GFP, an internal ribosomal entry site (IRES) and the reporter molecule, DsRED, which serves as the RTM transfection control (Figure 1). The *COL7A1*-minigene (*COL7A1*-MG) contains the 5' half of the reporter molecule GFP, followed by the RTM binding region, intron 102/exon 103 of *COL7A1*. Accurate *trans*-splicing leads to the fusion of both GFP split parts provided by the *COL7A1*-MG and the RTM via *trans*-splicing, manifested in the restoration and translation of full-length GFP (Figure 1). Co-transfection experiments of the *COL7A1*-MG together with individually selected RTMs into human embryonic kidney cells (HEK293 according to Murauer and colleagues provided useful information on the respective BD functionalities (data not shown) [10]. This result was represented by the intensity of the GFP signal and the number of GFP expressing cells analyzed by flow cytometry. With this, we selected an RTM (termed RTM_31), carrying a 108 bp BD complementary to the 5' part of intron 102 (from nt113 to nt220). This RTM was selected since it showed the highest *trans*-splicing efficiency amongst seven BDs tested in our screening system. Further, its target binding position within the 5' portion of intron 102 prevents interference with antisense oligonucleotides (AS RNAs), which bind to splicing elements and possible splicing regulators on the target molecule.

### 2.2. Selection of Antisense RNA Molecules (AS RNAs)

**Figure 2:**
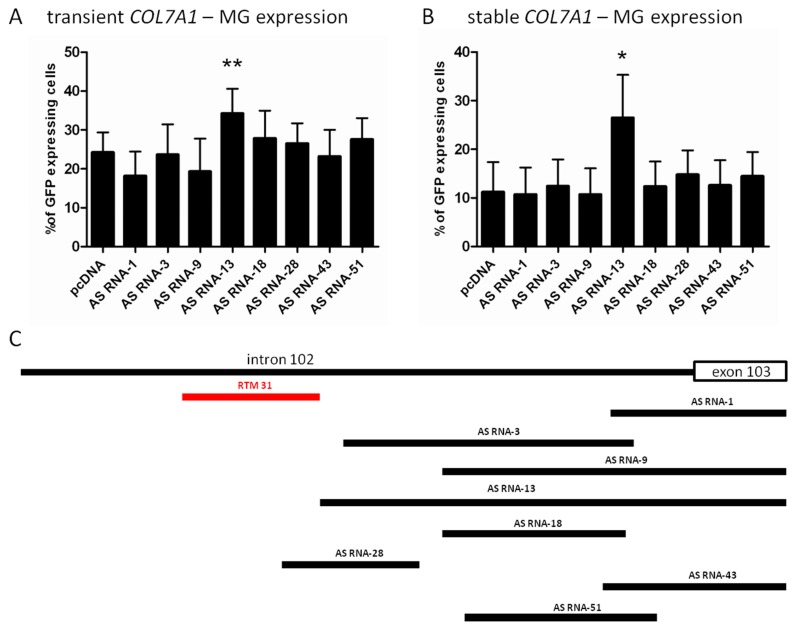
Comparison of AS RNA-mediated influence on *trans*-splicing efficiency. (**A**) Triple transfections of *COL7A1*-MG, RTM_31 and the most promising AS RNA (AS RNA-13) increased the amount of GFP expressing cells upon accurate *trans*-splicing from 24% to 34%. Mean + SD of five different experiments is shown. An unpaired T-test (two-tailed) was performed with GraphPad Prism software (GraphPad Software, San Diego, CA, USA) to prove the statistical significance between AS RNA-13 and the control pcDNA (** *p* value = 0.0073); (**B**) Co-transfection of RTM_31 and individual AS RNAs of the library into a *COL7A1*-MG expressing target cell line leads to a variable *trans*-splicing rate. AS RNA-13 showed the most promising effect, capable of increasing the *trans*-splicing efficiency of the RTM 2–3-fold on the protein level (measured by flow cytometry). AS RNA-13 was compared to the control (pcDNA). The mean + SD of 4 different experiments is shown. Unpaired, two-tailed *t*-test (* *p* value = 0.0298) confirmed statistical the significant increase in AS RNA-13 compared to the control pcDNA; and (**C**) Binding position of RTM_31 (red) and individual AS RNAs within the *COL7A1* target region.

Analogue to the BD selection, the fluorescence-based RTM screening procedure was performed in order to evaluate the efficiency of AS RNAs capable of masking competitive *cis*-splicing elements on the *COL7A1*-MG. In brief, the PCR amplified intron 102/exon 103 *COL7A1* region was fragmented by sonication and cloned into the pcDNA 4.0 vector. A powerful AS RNA will lead to an enhanced *trans*-splicing efficiency and increased full-length GFP expression in transfected HEK293 cells. In total, 86 different potential AS RNAs from the library were analyzed by sequencing. Eight individual AS RNAs (Figure 2) with antisense sequences for intron 102/exon 103 of *COL7A1* were selected for initial experiments. Triple-transfections of *COL7A1*-MG (0.5 µg), RTM_31 and selected AS RNAs in a relation of 1:6 (0.5 µg RTM: 3 µg AS RNA) into HEK293 cells resulted in a diverse level of GFP expression (the average amount of GFP expressing cells ranged from 18% to 34%) in comparison to cells transfected with RTM_31, *COL7A1*-MG and empty pcDNA 4.0 expression plasmids (Figure 2A). The most efficient AS RNA (AS RNA-13 with a binding length of 362 nucleotides) increased the level of GFP expressing cells from 24% (control) to over 34%. Endogenous experiments using a *COL7A1*-MG-expressing HEK293 target cell line confirmed the results of the triple transfection experiments. AS RNA-13 was able to increase the average % of GFP-expressing cells upon RTM_31 and AS RNA treatment from ~11% (pcDNA4.0) to over 26% (Figure 2B).AS RNA-13 probably blocks all potential splicing enhancer and splicing elements downstream of the RTM_31 binding region and is therefore a promising candidate for future experiments. Additionally, RTM_31 and AS RNA-13 have no overlapping binding sites within the target region, so we expect no significant binding competition and steric interferences between both molecules (Figure 2C).

### 2.3. AS RNA-13 Improves Trans-Splicing Efficiency in a Dose-Dependent Manner

**Figure 3:**
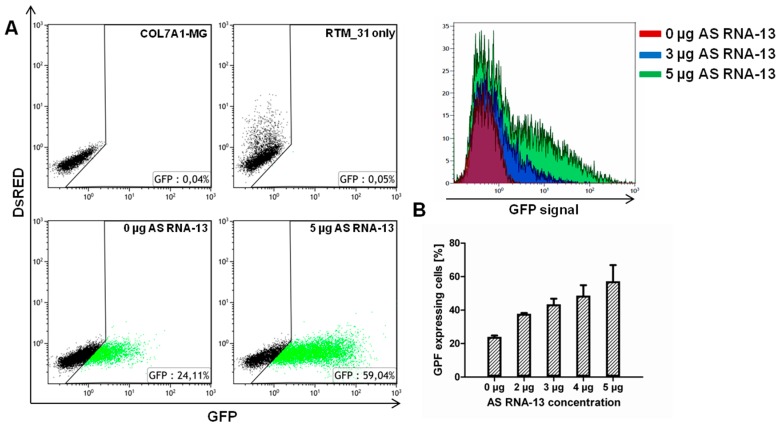
Increasing amounts of AS RNA-13 enhance GFP expression in a triple-transfection assay. After transfection of increasing amounts (0, 2, 3, 4 and 5 µg) of AS RNA-13 into HEK293 cells, co-transfected with 1 µg RTM_31 and 1 µg *COL7A1*-MG, the GFP-expressing cell population increased with the amount of AS RNA-13 introduced. (**A**) Dot blots showed increased GFP expression (green dots) in cells treated with 5 µg AS RNA-13 in comparison to the cell population exclusively transfected with RTM and *COL7A1*-MG-expressing plasmids; and (**B**) The increase of GFP expression correlates with the amount of AS RNA-13 (0 µg, 2–5 µg) transfected into HEK293 cells (the mean value + SD of two independent experiments is shown).

To prove these findings, a concentration dependent effect of AS RNA-13 on the *trans*-splicing efficiency was investigated by flow cytometric analysis after co-transfection of RTM_31, *COL7A1*-MG and AS RNA-13 into HEK293 cells 48 h post transfection. Here, the AS RNA-13 molecules induced an increment of GFP expression thereby reflecting the amount of *trans*-splicing events between RTM and target pre-mRNA molecules. The addition of 5 µg AS RNA-13 resulted in an obvious increase of GFP expressing cells, underlining the possibility to enhance the *trans*-splicing efficiency to significantly higher levels in co-transfection experiments (Figure 3A). Importantly, AS RNA-13 demonstrated its functionality also in a dose-dependent manner, showing: 23.47%, 34.91%, 40.22%, 44.30% and 51.61% of GFP expressing cells by the addition of 0, 2, 3, 4 or 5 µg AS RNA-13 plasmid respectively (Figure 3B). Both, the percentage of GFP expressing cells and the geometric mean of GFP expression was increased after AS RNA treatment. To reveal a similar plasmid level for each transfection, empty pcDNA 4.0 plasmids (Invitrogen, Carlsbad, CA, USA) were included for compensatory reasons.

### 2.4. Detection of Full-Length GFP by Western Blot Analysis

**Figure 4:**
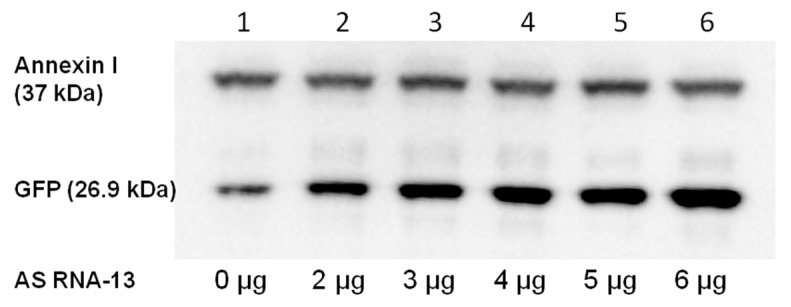
Detection of full-length GFP restoration by western blot analysis. The corresponding band for GFP is visible at a size of ~27 kDa on the nitrocellulose membrane after immunostaining. The amount of detected GFP increases with the amount of added AS RNA-13 (lanes 1–6: 0, 2–6 µg AS RNA-13). Analysis of the intensity of GFP signal revealed an up to eight-fold (relative quantification of GFP expression was measured using the Image Lab 3.0.1 software (Bio-Rad, Hercules, CA, USA) up-regulation after the addition of 6 µg AS RNA-13 expressing plasmids. Annexin I was included as loading control in the experiment visible at a size of 37 kDa on the nitrocellulose membrane.

To confirm the accurate fusion of the two GFP split parts by RNA *trans*-splicing on protein level, we performed western blot analysis 48 h post-transfection. We detected full-length GFP (26.9 kDa), resulting from successful *trans*-splicing. As shown in Figure 4, the intensity of the full-length GFP band was much higher after AS RNA-13 administration. This experiment stressed the accurate fusion of both GFP split parts by RNA *trans*-splicing and underlined the possibility to enhance the *trans*-splicing efficiency by the addition of AS RNA-13, which probably interferes with *cis*-splicing elements on the *COL7A1*-MG.

### 2.5. Trans-Splicing Efficiency Analysis by Semiquantitative Real Time PCR (sqRT-PCR)

**Figure 5:**
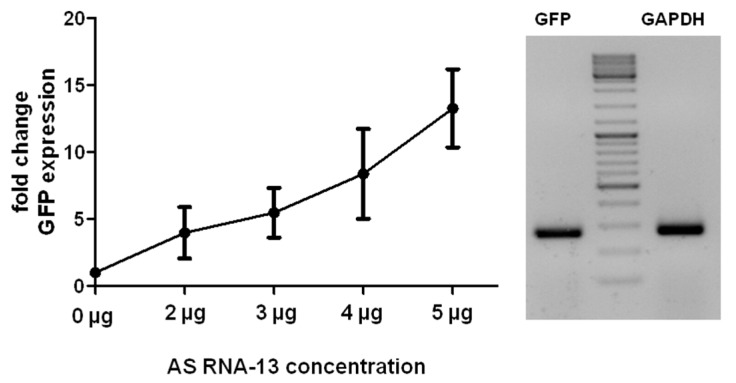
Quantification of full-length GFP transcripts by sqRT-PCR. Co-transfection of AS RNA-13, RTM_31 and *COL7A1*-MG-expressing plasmids into HEK293 cells increased the level of *trans*-splicing. The amount of GFP transcripts, representing accurate *trans*-splicing events between RTM_31 and *COL7A1*-MG, was quantified by sqRT-PCR using GFP specific primers. Similarly to the data obtained by flow cytometric measurements (Figure 3A) the GFP expression correlates with the amount of given AS RNA-13. The more AS RNA-13 expression plasmids were introduced into the cells, the higher was the *trans*-splicing efficiency manifested in the expression of full-length GFP transcripts. The mean value ± SD of three independent experiments is shown. All values were normalized to GAPDH.

The influence of AS RNA-13 on the *trans*-splicing efficiency of the designed *COL7A1*-MG and RTM_31 was further analyzed by sqRT-PCR (Figure 5). There, the relative levels of total full-length GFP transcripts were analyzed in co-transfected cells. Experiments showed an increase of GFP expression, when AS RNA-13 was additionally provided. This confirms the data obtained by flow cytometric analysis and western blot analysis (Figure 3 and Figure 4). More precisely, treating HEK293 cells with AS RNA-13 resulted in an up to 12-fold increase of full-length GFP transcripts over cells exclusively treated with RTM_31 and empty pcDNA 4.0 expression plasmids (Figure 5).

## 3. Discussion

RNA* trans*-splicing has been described as an elegant tool to reprogram mRNAs with a wide range of applications. Although RNA *trans-*splicing can be applied e.g., to induce toxin-mediated cell death in tumour cells [18,19], the technique is predominantly used in RNA therapy approaches for monogenic diseases [1,2,6]. Whereas conventional full-length cDNA gene therapy strategies are often limited by the packaging size of viral vectors, the size of RNA *trans*-splicing molecules (RTMs) is drastically reduced, facilitating the production of viral particles for delivery into target cells. Additionally, the RTM-induced *trans-*splicing reaction is under endogenous control decreasing common issues in gene therapy, like over- and ectopic expression of the transgene [6,20,21].

Besides RTM design in general, the type of mutation has a great influence on the outcome of the RTM treatment. Individuals, carrying a heterozygous nonsense mutation in the *COL7A1* gene, are phenotypically healthy. Therefore the amount of type VII collagen needed to prevent blistering lies between 0% and 50% [22]. Additionally, 10% of normal type VII collagen levels were shown to be sufficient to reduce the severity of the phenotype of transgenic mice [23]. Therefore in recessive dystrophic epidermolysis bullosa (RDEB) a relatively low level of type VII collagen expression may be sufficient for the restoration of anchoring fibrils within the basal membrane zone of the skin. In a series of experiments Murauer *et al.*, have shown that *trans*-splicing into the *COL7A1* pre-mRNA produces sufficient amounts of protein to restore the phenotype of patient keratinocytes [5]. Type VII collagen was expressed after 3' RNA *trans*-splicing leading to a normal localization of the protein at the basement membrane zone in skin equivalents. In this study single clonal cells were isolated of the RTM transduced and antibiotic-selected RDEB keratinocytes. The most promising clone of all RTM treated RDEB keratinocytes synthesized and secreted full-length type VII collagen protein in an amount comparable to that of normal human keratinocytes.

However, due to the wide spectrum of clinical phenotypes in EB underlying also dominant negative mutations [24], probably higher *trans*-splicing mediated repair efficiencies are required in order to significantly reduce the dominant negative protein expression. Cao* et al.*, developed a mouse model for EBS. By increasing the ratio of wildtype to mutated polypeptides they were able to improve the phenotype [25]. A partial reversion of the phenotype in EBS* in vitro* by RNA *trans*-splicing was already shown for the keratin 14 gene [4]. Due to the possibility of converting mutated to wildtype transcripts, the technology of RNA *trans*-splicing can be well suited for the treatment of dominantly inherited disorders.

The RNA *trans*-splicing efficiency depends on the binding position, length and GC content of the RTM’s binding domain (BD). Especially RTMs binding in close proximity to the competitive target *cis*-splice site show enhanced *trans*-splicing efficiencies [5,9,10]. Additionally, it was recently demonstrated in a spinal muscular atrophy (SMA) model that the co-expression of an antisense oligonucleotide (AS RNA) that masked the downstream competing 3' *cis*-splice site could significantly improve the efficiency of the *trans*-splicing reaction induced by a given RTM [13,14].

In this manuscript we evaluated the efficiency of randomly designed AS RNAs capable of masking competitive *cis*-splicing elements on a *COL7A1*-minigene, comprising intron 102 and exon 103 of the *COL7A1* gene, in order to increase the efficacy of a previously identified functional RTM, termed RTM_31. Using our fluorescence-based screen for RTM-BD selection we showed the possibility to identify an AS RNA capable of enhancing the efficiency of *trans*-splicing manifested in an enhanced GFP expression. The screening system will further enable us to define molecular determinants of efficient AS RNA function, including optimal length and location. Since RNA splicing is important in the regulation of gene expression and impacts disease pathogenesis, much effort is being put into deciphering the “splicing code”. Our established AS RNA screening system has the potential to impact the field of RNA biology by contributing to our understanding of the factors that influence splicing. Especially the identification and characterization of novel splicing regulatory elements (SREs) will increase the knowledge in this field.

To further confirm our findings on endogenous level in patient cells the most efficient BD-AS RNA combination will be cloned into a viral vector of choice carrying the wildtype coding region of *COL7A1* to be introduced. The co-administration of both interfering RNA molecules will help to increase gene correction by RNA *trans*-splicing thereby facilitating phenotypic correction of the disease-associated phenotype in EB. This will enable us to move forwards to the clinical development and application of this therapeutic strategy in EB patients, which can also be used for any other monogenetic disease.

## 4. Materials and Methods

### 4.1. Construction of Screening Plasmids

Screening constructs were designed and cloned according to the protocol of Bauer* et al.* [12]. For cloning of the target region intron 102/exon 103 of *COL7A1* into the *COL7A1*-minigene (*COL7A1*-MG) expression vector an intron 102 specific forward primer (5'-GATCGATATCACAGGGAAGAGGGTGGTGTACTCTC-3'), an exon 103 specific reverse primer (5'-GATCGCGGCCGCCTGGGGTCCCAGGAGTCCACG-3') and genomic DNA from a healthy donor were included in the PCR. The PCR product was cloned into the screening vector using the restriction sites for EcoRV and NotI. Gel-extractions of amplified PCR products were performed using a GFX™ PCR DNA and Gel Band Purification Kit (GE Healthcare, Chalfont St. Giles, UK). Plasmid preparations were carried out using a Plasmid Mini Prep Kit (Sigma–Aldrich, Taufkirchen, Germany), according to the manufacturer’s protocol. Sequence analysis of all plasmids and PCR products was performed using a 3500 ABI automated sequence analyzer and ABI PRISM dye terminator cycle sequencing kit (Applied Biosystems, Foster City, CA, USA).

### 4.2. Cloning of COL7A1-Minigene PiggyBac (PB) Transposon Cassette

For cloning of the 5' part of GFP and the *COL7A1* region intron 102/exon 103 into the PB transposon vector (System Biosciences, Mountain View, CA, USA) a 5' GFP specific forward primer (5'-GATCGGATCCCACCATGGTGAGCAAGGG-3'), and an exon 103 specific reverse primer (5'-GATCGCGGCCGCCTGGGGTCCCAGGAGTCCACG-3') were used to amplify the PCR product from the target screening plasmid described above. The PCR product was cloned into the transposon expression vector using the restriction sites for BamHI and NotI.

### 4.3. Development of Stable COL7A1-Minigene Expressing HEK293 Cell Line

For the generation of a cell line, stably expressing *COL7A1*-MG, we use the HEK293 cell line (Stratagene, La Jolla, CA, USA) which does not express *COL7A1*. For this, the target transposon vector was co-transfected with a PiggyBac transposase expression vector (System Biosciences) into the cell line using the JetPEI reagent (Polyplus-transfection SA, Illkrich, France) according to the manufacturer’s protocol. Recognition of the inverted repeats by the PiggyBac transposase induced the integration into the host genome. Subsequent antibiotic selection with puromycin led to target expression in the majority of remaining cells, which was verified by fluorescence-activated cell sorting (FACS) analysis. Full-length integration of the *COL7A1*-MG into the cell’s genome was further detected by target-specific PCR amplification from genomic DNA, isolated from the transfected cells.

### 4.4. Construction of Antisense Oligonucleotide (AS RNA) Library

An AS RNA library was constructed by the fragmentation of the PCR amplified intron 102/exon 103 region of *COL7A1* by sonication. The resulting fragments were cloned into a pcDNA 4.0 plasmid (Invitrogen) using the restriction site for EcoRV and analyzed for their right orientation (complementary to the target region) by sequence analysis.

### 4.5. Cell Culture and Plasmid Transfection

For co-transfection studies the human embryonic kidney cell line HEK293 (Stratagene) was utilized and grown in Dulbecco’s Modified Eagle Medium DMEM supplemented with 10% Fetal Bovine Serum (FBS) and 100 U/mL penicillin/streptomycin (Biochrom, Berlin, Germany) at 37 °C and 5% CO_2_ in a humidified incubator. Passaging of the cells was carried out by Trypsin-EDTA (Biochrom) treatment following centrifugation at 250× *g* for 5 min. Pelleted cells were seeded in new tissue culture plates (60 mm). Transient transfections of screening plasmids were performed the next day using the jetPEI reagent (Polyplus-transfection SA) according to the manufacturer’s protocol.

### 4.6. RNA Isolation and cDNA Synthesis

HEK293 cells were harvested 48 h post co-transfection and RNA was isolated using the RNeasy Mini Kit (Qiagen, Valencia, CA, USA) according to the manufacturer’s protocol. One microgram of RNA was digested with DNAse I for 30 min at room temperature. Afterwards cDNA synthesis was performed with iScript™ cDNA Synthesis Kit (Bio-Rad) according to the manufacturer’s protocol.

### 4.7. SqRT-PCR

SqRT-PCR was performed to detect full-length GFP fusion transcripts in treated HEK293 cells. For PCR analysis, a 5' GFP-specific forward primer (5'-GCTGACCCTGAAGTTCATCTG-3'), a 3' GFP-specific reverse primer (5'-GGTCAGCTCGATGCGATTCACC-3'), cDNA of treated cells and GoTaq^®^ qPCR Master Mix (Promega, Madison, WI, USA) were included. The PCR was performed using a Bio-Rad CFX™ system with the following conditions: 95 °C for 2 min, and 40 cycles of 20 s at 95 °C, 20 s at 66 °C, and 20 s at 72 °C. The experiment was carried out in duplicates and repeated two times. Correct PCR products were verified by direct sequence analysis.

### 4.8. Flow Cytometric Analysis

The amount of *trans*-splicing events, manifested in the expression of GFP, was measured 48–96 h after transfection using a Beckman Coulter FC-500 FACS analyser (Beckman Coulter, Vienna, Austria). The transfected HEK293 cells were washed once with phosphate buffer saline (Dulbecco’s PBS) and detached from the cell culture plates using trypsin-EDTA. Trypsin-EDTA was inactivated with Solution A (7.25 g HEPES; 1.8 g glucose; 0.22 g NaCl; 0.27 g NaHPO_4_; 1 mL phenolred; 900 mL H_2_O; pH 7.4) supplemented with 10% FBS. After centrifugation for 5 min at 250× *g*, 15,000 cells were analyzed according to their GFP expression.

### 4.9. Western Blot Analysis

*Trans*-splicing induced GFP expression was detected on the protein level via western blot analysis 48–96 h post transfection. HEK293 cells were washed once with PBS buffer, harvested and resuspended in radioimmunoprecipitation assay buffer RIPA buffer (Santa Cruz Biotechnology, Heidelberg, Germany). Samples were denatured for 5 min at 95 °C in 4× loading buffer (0.25 M Tris-HCl; 8% SDS; 30% glycerol; 0.02% bromphenol blue; 0.3 M β-mercaptoethanol; pH 6.8). SDS-gel-electrophoresis was performed at 100 Volt for 2 h using a NuPAGE 4%–12% Bis-Tris gel (Invitrogen) and NuPAGE MOPS running buffer. Proteins were electro-blotted onto a nitrocellulose membrane (Amersham Hybond-ECL, Amersham, Buckinghamshire, UK) at 0.25 A for 75 min. The membrane was blocked with Tris buffered saline (TBS) + 5% milk powder for 1 h at RT and incubated with a primary anti-GFP rabbit IgG antibody (Biomedica, Vienna, Austria) in a dilution of 1:1000 in TBS + Tween (0.2%) including 5% BSA at 4–8 °C over night. After three washing steps (3 × 15 min) with TBS-T (0.2%) the membrane was incubated with a secondary HRP Envision + labelled anti rabbit antibody (1:200 in TBS-T + 5% milk powder, Dako, Vienna, Austria) and incubated for 2 h at room temperature. Finally, GFP expression was visualized using the Immun-Star Western C Kit (Bio-Rad). Anti-Annexin I mouse monoclonal IgG antibody (1:2000 in TBS-T, Santa Cruz) and a secondary HRP Envision+ labelled anti mouse antibody (1:200 in TBS-T Tween, Dako) were used to detect human Annexin I which served as control for protein loading. The relative quantification of GFP expression was measured using the Image Lab 3.0.1 (Bio-Rad) software.

## 5. Conclusions

Within this study we report for the first time, that the combined application of RNA *trans*-splicing molecules (RTM) and antisense oligonucleotides (AS RNAs) can significantly increase the *trans*-splicing efficiency of a selected RTM specific for the EB-associated gene *COL7A1*. The important innovative aspect of this study is the establishment of an unbiased RTM and AS RNA screening method that can be used not only for the improvement of *COL7A1* repair, but also for other genes affected in EB as well as other genetic diseases. Our screening system is further applicable to select efficient molecules for use in any antisense-based therapy. This study provides evidence that the use of AS RNAs significantly modulates the *trans*-splicing reaction by blocking the recognition of splice sites or masking exonic signals, which leads to increased expression of the corrected transcript. Therefore this approach can be considered as a promising strategy for the correction of inherited monogenic disorders.
